# Heat exposure as a cause of injury and illness in mine industry workers

**DOI:** 10.1093/annweh/wxae011

**Published:** 2024-03-04

**Authors:** Sarah M Taggart, Olivier Girard, Grant J Landers, Karen E Wallman

**Affiliations:** School of Human Sciences (Sport Science, Exercise and Health), The University of Western Australia, 35 Stirling Highway, Crawley, WA 6009, Australia; School of Human Sciences (Sport Science, Exercise and Health), The University of Western Australia, 35 Stirling Highway, Crawley, WA 6009, Australia; School of Human Sciences (Sport Science, Exercise and Health), The University of Western Australia, 35 Stirling Highway, Crawley, WA 6009, Australia; School of Human Sciences (Sport Science, Exercise and Health), The University of Western Australia, 35 Stirling Highway, Crawley, WA 6009, Australia

**Keywords:** dehydration, heat exposure, heat-related illness, injury, mining industry

## Abstract

The objective of this study was to explore the association between ambient temperature and injuries and illnesses experienced by mine industry workers. Eleven years of de-identified data from a mine industry company in Australia was explored in regards to injuries and illnesses occurring due to outdoor exposure. Each case was filtered for reported symptoms, and meteorological data to match the location of the mine site and date reported were sourced. Of the 18 931 injuries and illnesses observed over the 11-year period, 151 cases of heat-related illness due to outdoor exposure were reported. Twenty-five conditions/symptoms of heat-illness were found, with the most prevalent being dehydration (*n* = 81), followed by heat rash (*n* = 40), dizziness (*n* = 24), and headache (*n* = 23). The mean number of symptoms reported by each worker was 2 ± 1. There was a positive correlation between ambient temperature and injuries/illnesses (*r*^2^ = 0.89, *P* < 0.001), where, as temperature increased so did the number of reported heat-related illnesses. Underreporting of heat-related illness and injury in the mining industry is likely, which is a risk to the health and wellbeing of employees. Workers require industry specific training about the severity of heat stress and the associated prevention strategies.

**What’s Important About This Paper?**

Among Australian miners, there is a positive correlation between ambient temperature and injuries and illnesses reported due to environmental conditions, including heat-related illness. There is a need to ensure workers remain hydrated and recognize the signs and symptoms of heat-related illness so as to minimize risks.

## Introduction

The prevention of workplace injuries and illnesses is of great importance for the health and wellbeing of workers, as well as for economic gain. Workplace injuries and illnesses have important financial implications for the economy, including health care costs, the effects of absenteeism (productivity loss due to time off work), and presenteeism (reduced productivity at work; Safe Work Australia, 2022).

The risk of experiencing a workplace injury or illness is higher in hotter working environments (Varghese et al. 2019). Working in the heat can overwhelm the body’s physiological cooling mechanisms, leading to increased core temperature, heart rate and thermal discomfort levels, and greater dehydration (Gaoua et al. 2011, 2012; Périard and Racinais 2019). The negative consequences of heat exposure include deteriorated cognitive performance (Mazloumi et al. 2014) and increased fatigue, which together or separately can impair physical performance and productivity (Foster et al. 2021).

Prolonged heat exposure can result in numerous heat-related illnesses (Leyk et al. 2019). Specifically, heat cramps and heat rash represent mild forms of heat illnesses, usually occurring after prolonged activity in the heat. These heat complications can be treated with adequate hydration and by moving to a cooler environment (Gauer and Meyers 2019). Heat syncope and heat exhaustion, often characterized by symptoms of light-headedness, dizziness, thirst, headache, fatigue, weakness, and high core temperature are categorized as moderate heat illnesses. If left untreated, they can progress to a more severe condition called heat stroke (Gauer and Meyers 2019). Heat stroke is characterized by a core temperature >40.5 °C and can result in central nervous system dysfunction. Common symptoms presenting are seizures, altered mental status, tachycardia, and eventually coma (Gauer and Meyers 2019).

Studies investigating the relationship between environmental temperature and injury and illness rates describe various relationships, including a *J-*shaped curve, an inverted *U-*shaped curve or a linear curve (Xiang et al. 2014b; McInnes et al. 2017; Varghese et al. 2019) The disparities in these relationships may stem from the broad range of workers included in these studies, variations in locations, and/or differences in the reporting competence of injuries and illnesses in different workplaces.

This study aimed to explore the association between outdoor ambient temperature and injuries and illnesses experienced in mining workers over an 11-year period in a mining industry company in Australia.

## Materials and methods

### Data collection

De-identified data of 18 931 incidence of injury and illness were reported by an Australian mining company from March 2012 to March 2023. On average, the company operates at ~250 mine sites and deploys ~5000 people to these sites per year. Whenever employees experienced symptoms of illness or an injury, they verbally reported them to the site manager or safety member on-site, who inputted the information into a database. Each incident included information regarding site name, date, a description of the event, agency (direct cause of injury/illness), mechanism (how the injury/illness was sustained), nature (type of injury/illness), and the injured body part. Optional fields included the number of days into the swing, length of employment, occupation, and roster type. Corresponding maximum temperatures reported on the day of each incident were sourced from the closest weather station, as provided by the Bureau of Meteorology. Dehydration cases were usually diagnosed after providing a urine sample to the site medic, who assessed for urinary specific gravity. Brief descriptions of each incident were carefully screened in the current study to identify key symptoms of heat illness, with a list of frequently reported symptoms compiled.

Data were initially screened for the agency of injury using the phrase *“outdoor environment”* (*n* = 1128). Data were then filtered so that the mechanism of injury was further defined by using the phrases *“due to exposure to radiation”* or *“contact or exposure to heat”* (*n* = 94). All other cases within the dataset were then screened using key symptoms of heat-related illnesses. This additional screening led to the inclusion of 57 cases. In total, this resulted in 151 incidents. These incidents encompassed eight broad occupations, including 122 cases from service workers (e.g., housekeepers, kitchen hands, and mine-site cleaners), 6 from chefs, 9 from maintenance workers (gardeners, groundsmen, plumbers, electricians), 3 from administrative workers and supervisors, 2 from drivers and security officers, 1 being a health and wellbeing coordinator, while 3 worked in occupations that were not reported. Ethics approval was granted by the University of Western Australia (2023/ET000272).

### Data analysis

Data is presented as counts or mean ± SD. Figures were created using Prism (version 5) and Tableau Desktop (version 2023.2). A Pearson’s correlation analysis was used to assess the relationship between illnesses and maximum temperature (R studio). Counts were calculated on a monthly basis, combining data from all years (e.g., April: *n* of 5 = 2013 + 2014 + 2016 + 2019 + 2020), while the mean maximum temperature for each month was used in the correlation analysis. Total number of employees working on site over the summer months (October, November, December, January, February, and March) and winter months (April, May, June, July, August, and September) were considered, with data showing that average employees on site in winter was 51% and 49% in summer.

## Results

**Fig. 1.**
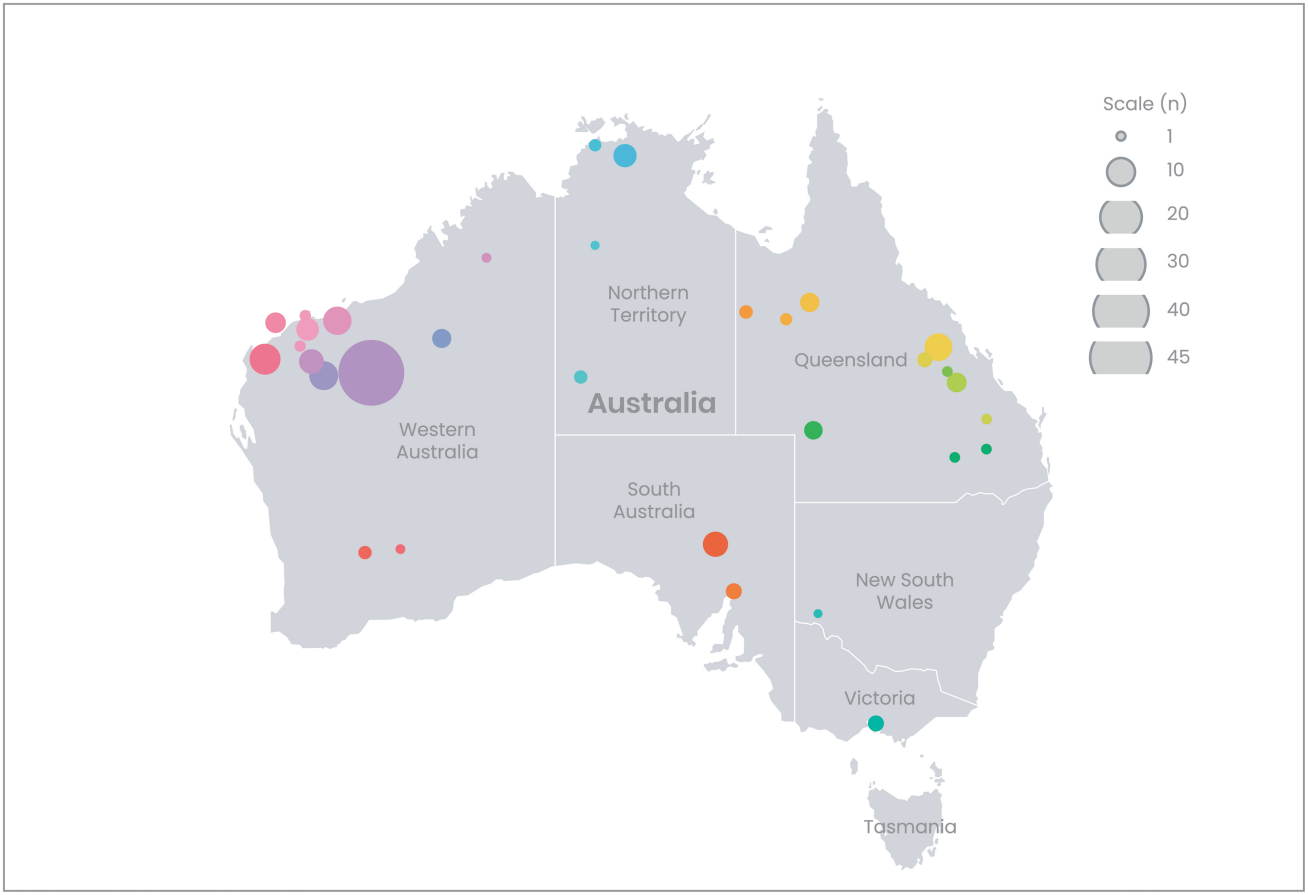
Distribution of reported injuries and illnesses. Point colours differentiate site locations.

The initial screening of data showed a trend where the total number of reported injuries and illnesses (e.g., heat related illness, strains, falls, trips etc.) occurring due to outdoor environmental conditions was greater during summer than winter months (*n* = 1128). Upon filtering data to include only heat-related illnesses/injuries, it was found that 151 incidents occurred at 78 different mine sites, distributed across 32 postal code areas (Fig. 1). Specifically, there were 95 incidents in Western Australia, 29 in Queensland, 11 in South Australia, 10 in Northern Territory, 3 in New South Wales, and 3 with unknown locations. The number of injuries reported within each quartile of a roster length are reported in Table 1. Length of employment varied with 36% of workers employed for <1 month, 18% employed for 1–3 months, 22% for 4–12 months, and 23% employed for >1 y.

**Table 1.** The distribution of injuries and illnesses that occurred during each roster type, categorized into the quartile in which they occurred relative to the length of a swing (*n* = 101). The percentage value is relative to the proportion of workers in each quartile for that roster type.

Roster type	First quartile *n* (%)	Second quartile *n* (%)	Third quartile *n* (%)	Fourth quartile *n* (%)	Grand Total
6 days on: 1 day off		1 (100)			1
7 days on: 7 days off	1 (20)	1 (20)	2 (40)	1 (20)	5
8 days on: 6 days off			1 (100)		1
9 days on: 5 days off	2 (15)	2 (15)	4 (31)	5 (39)	13
14 days on: 7 days off	22 (32)	20 (29)	15 (22)	12 (17)	69
14 days on: 14 days off	1 (33)	2 (67)			3
28 days on: 7 days off	2 (29)		3 (42)	2 (29)	7
28 days on: 28 days off	1 (50)		1 (50)		2

There were a total of 25 different symptoms/conditions experienced. The most commonly reported symptoms/conditions were dehydration (*n* = 81), followed by heat rash (*n* = 40), dizziness (*n* = 24), and headache (*n* = 23). On average, each individual reported 2 ± 1 symptoms.

**Fig. 2.**
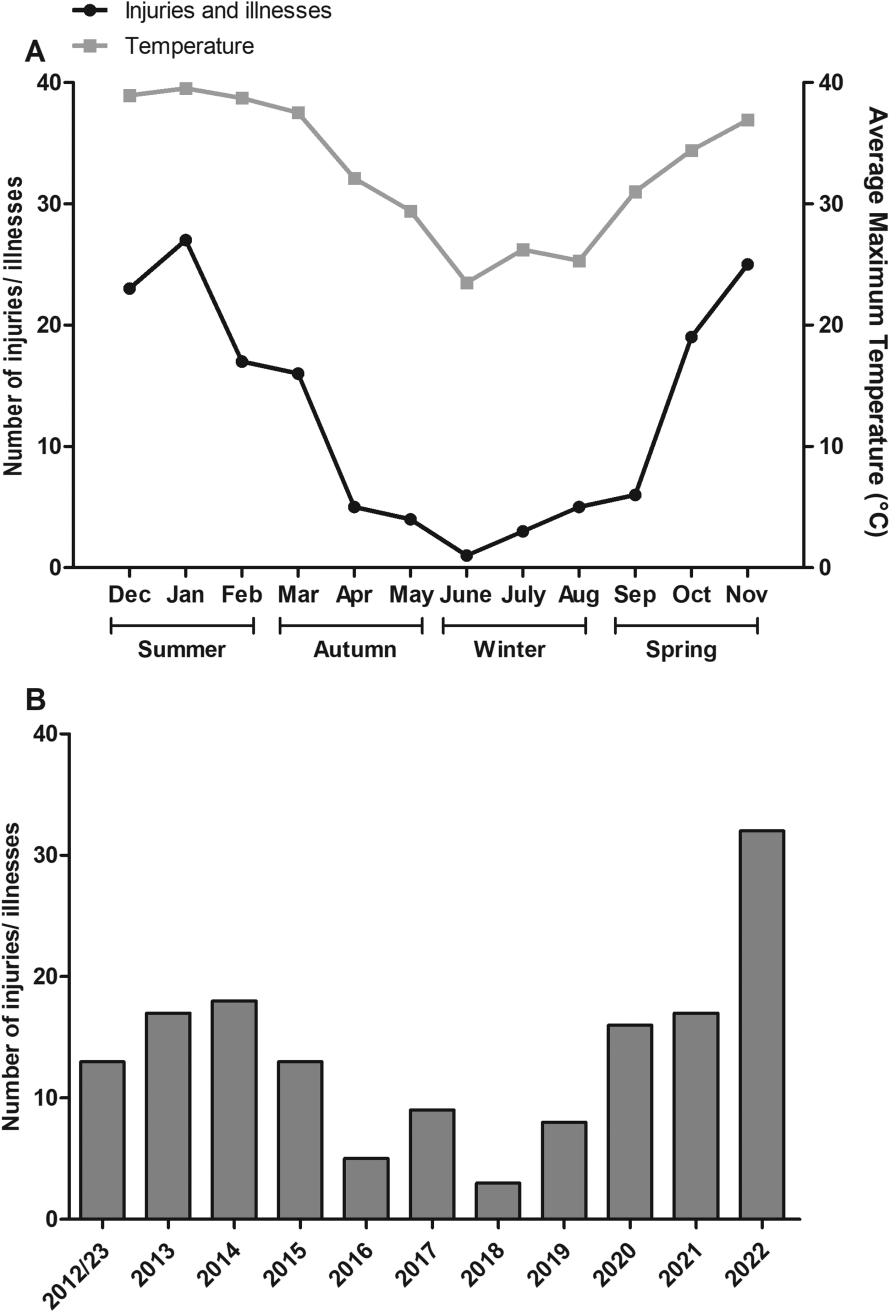
Number of heat-related injuries and illnesses monthly (A) and yearly (B). For Panel A the number of heat-related injuries and illnesses that occurred appear in black and average maximum temperature is grey. In Panel B the injuries and illnesses experienced in 2012 and 2023 are combined due to the data starting in March 2012 and end in March 2023.

The frequency of injuries followed a linear trend, indicating that fewer injuries occurred in the work environment during cooler compared to hotter days (*r*^2^ = 0.89, *P* < 0.001). Figure 2A shows the monthly trend, with peaks in the number of incidents recorded during the summer months and lows during the winter months, with the per yearly trend shown in Fig. 2B.

## Discussion

This study demonstrated a positive linear relationship between ambient temperature and the number of injuries and illnesses experienced due to outdoor exposure in mine service workers over an 11-year period. In total, 1,128 injuries and illnesses occurred due to outdoor environmental temperature, with 151 being directly due to heat-related injury/illness. Dehydration, headache, heat cramps, and dizziness were the most prevalent symptoms. These symptoms are similar to those reported by underground mining workers (Donoghue et al., 2000). Donoghue et al. (2000) also reported that worker’s mean urinary specific gravity value was 1.027, representing a classification of “significantly dehydrated’”(Casa et al. 2000). Many of these early symptoms of heat exhaustion may seem “usual” and considered to be of minimal harm to a worker during or after a day of physical work in the heat and more often than not go unreported (Xiang et al. 2014a). The symptoms and severity of heat exhaustion need to be clearly understood by workers and employers so that the health and wellbing of staff are managed appropriately.

At least half of the heat-related illnesses reported in the current study were experienced by people that had been employed for less than 1 year. Newly employed workers may not have yet received adequate education around the risks of heat illness and the prevention strategies that can be utilized, hence putting these workers at a higher risk. Notably, Bonafede et al. (2022) conducted a questionnaire assessing workers’ perception of heat stress in Italy. Only one in four employees had received training regarding the prevention of heat stress, and only 17.1% received warnings when ambient temperatures were high, with this resulting in an elevated risk of experiencing a heat-related illness. Additionally, workers lacked precise knowledge regarding the risks associated with heat exposure (Bonafede et al. 2022). Therefore, it is important for workplaces to educate all workers of the symptoms and severity of heat illnesses, as well as prevention measures. This training should be part of an induction process and be repeated regularly over time.

In the current study, as temperatures increased so did the number of heat-related injuries and illnesses, as seen by a positive linear trend (*r*^2^ = 0.89, *P* < 0.001). This supports current literature by McInnes et al. (2017) and Adam-Poupart et al. (2015) that described a positive linear relationship between workplace incidents and maximum daily temperature, where the frequency of heat-related incidents increased with rising temperatures. Their findings differed from other studies that reported an inverse *U-*shaped relationship, where the number of injuries and illnesses increased with rising ambient temperature until reaching a certain point (~14.2 °C and 37.7 °C), after which the number declined (Morabito et al. 2006; Xiang et al. 2014b). This decline was likely due to workplace safety restrictions designed to protect workers from heat stress by either requiring the cessation of work at extreme temperatures or the implementation of stricter work rest schedules. Varghese et al. (2019) reported a *J*-shaped relationship between temperature and workers’ compensation claims. They found that compared to temperate environmental conditions (25 °C), the relative risk of suffering from an injury or illness increased to 1.08 in moderate temperatures (33.3 °C), and 1.30 in extreme temperatures (40.6 °C). The results of the current study do not align with the inverted *U* or *J-*shaped relationship described above. The discrepant findings between these studies and the current one may relate to the exclusion of winter data (*U-*shaped curve), the larger sample sizes or the use of workers compensation data in these studies. These factors, individually or collectively, could have influenced the shape of the observed relationship between temperature and the number of injuries and illnesses recorded. Further research should compare injuries and illnesses, specifically due to heat exposure, to determine if an inverted *U*, *J-*shaped or linear relationship exists when cooler temperatures are taken into consideration.

The number of injuries and illnesses experienced due to outdoor conditions in this study averaged ~100 per year. However, the total number (*n* = 151) of specifically heat-related illnesses experienced appears relatively small over an 11-y period. It should be noted that heat-related illnesses in the workplace are often under-reported (Xiang et al. 2014a) due to several reasons. First, workers may lack awareness or education about heat-related illness symptoms, hindering recognition and reporting (Han et al. 2021). Second, some employees may not know how to report injuries, fear marginalization, or face reporting obstacles (Kyung et al. 2023). Third, non-urgent cases may be excluded from reporting, and managers might monitor acute symptoms to avoid official records. Lastly, heat exposure can mask or misclassify heat-related illnesses in the presence of pre-existing conditions (Kenny et al. 2010; Xiang et al. 2014a).

The study’s limitations include a small sample size, based on records from a single company and variability in symptoms and description of injuries and illnesses, stemming from the lack of specific questions in the reporting system. Relative humidity data were not available, a limitation since high relative humidity can impede sweat evaporation, potentially increasing body temperature and the risk of heat illnesses (Périard and Racinais 2019). Lastly, the misreporting of incident times and inaccessibility of demographic data limit comprehensive data interpretation.

## Conclusion

There was a significant association between outdoor temperature exposure and the frequency of both general injuries and illnesses, as well as heat-related illnesses in the workplace. It is crucial to provide workers with industry-specific training and education about the severity of heat stress and effective prevention strategies. Practically, workers should complete urine colour checks over their swing to ensure they are consuming enough fluids to avoid dehydration. Implementing measures such as alerts from managers can further enhance workplace safety in hot environments, reducing the risk of injuries and illnesses.

## Data Availability

The data underlying this article cannot be shared due to the legal restrictions from the Company in which the data was source from.
